# Mechanical Circulatory Support Systems in Fulminant Myocarditis: Recent Advances and Outlook

**DOI:** 10.3390/jcm13051197

**Published:** 2024-02-20

**Authors:** Max Lenz, Konstantin A. Krychtiuk, Robert Zilberszac, Gottfried Heinz, Julia Riebandt, Walter S. Speidl

**Affiliations:** 1 Department of Internal Medicine II, Division of Cardiology, Medical University of Vienna, 1090 Vienna, Austriawalter.speidl@meduniwien.ac.at (W.S.S.); 2 Ludwig Boltzmann Institute for Cardiovascular Research, Waehringer Guertel 18-20, 1090 Vienna, Austria; 3 Department of Cardiac Surgery, Medical University of Vienna, 1090 Vienna, Austria

**Keywords:** mechanical circulatory support, MCS, fulminant myocarditis, ECMO, VAD

## Abstract

**Background:** Fulminant myocarditis (FM) constitutes a severe and life-threatening form of acute cardiac injury associated with cardiogenic shock. The condition is characterised by rapidly progressing myocardial inflammation, leading to significant impairment of cardiac function. Due to the acute and severe nature of the disease, affected patients require urgent medical attention to mitigate adverse outcomes. Besides symptom-oriented treatment in specialised intensive care units (ICUs), the necessity for temporary mechanical cardiac support (MCS) may arise. Numerous patients depend on these treatment methods as a bridge to recovery or heart transplantation, while, in certain situations, permanent MCS systems can also be utilised as a long-term treatment option. **Methods:** This review consolidates the existing evidence concerning the currently available MCS options. Notably, data on venoarterial extracorporeal membrane oxygenation (VA-ECMO), microaxial flow pump, and ventricular assist device (VAD) implantation are highlighted within the landscape of FM. **Results:** Indications for the use of MCS, strategies for ventricular unloading, and suggested weaning approaches are assessed and systematically reviewed. **Conclusions:** Besides general recommendations, emphasis is put on the differences in underlying pathomechanisms in FM. Focusing on specific aetiologies, such as lymphocytic-, giant cell-, eosinophilic-, and COVID-19-associated myocarditis, this review delineates the indications and efficacy of MCS strategies in this context.

## 1. Introduction

Myocarditis is a disease characterised by myocardial inflammation. It can be caused by various underlying conditions, including infections, autoimmune diseases, exposure to chemicals, and adverse drug reactions [1]. Symptoms can range from mild discomfort, chest pain, and fatigue to severe complications like heart failure (HF), arrhythmias, or sudden cardiac death [2]. Fulminant myocarditis (FM) constitutes a severe and life-threatening form of acute cardiac injury and is characterised by rapidly progressing myocardial inflammation, leading to significant impairment of cardiac function. FM is often associated with haemodynamic instability, leading to impaired organ perfusion and subsequent multiorgan failure, a syndrome termed cardiogenic shock [3]. This review summarises the available evidence regarding the indications and efficacy of mechanical circulatory support (MCS) systems, like venoarterial extracorporeal membrane oxygenation (VA-ECMO), microaxial flow pumps, and ventricular assist devices (VADs), in critically ill patients suffering from FM. Moreover, our goal is to shed some light on important aspects such as ventricular unloading and previously proposed weaning approaches. In addition to general recommendations, differences in the underlying pathomechanism of FM and their implications for MCS are highlighted. Particular emphasis is put on the described histological subtypes, and support strategies are outlined in these distinct contexts. Our aim is to provide a comprehensive review of FM and MCS by presenting contemporary evidence and spotlighting potential future developments in the field.

## 2. Materials and Methods

A systematic literature review for articles published until January 2024 was performed by the authors, including PubMed, Web of Science, and Google Scholar. A comprehensive search strategy was executed and included the following keywords: acute myocarditis, fulminant myocarditis, acute heart failure, mechanical circulatory support, ECMO, Impella, VAD, and COVID-19. A total of 142 articles were deemed relevant within the scope of this review. The publications were categorised into thematic clusters and analysed according to their relevance within the respective field. Although the available body of evidence includes paediatric and adult data, we focused primarily on the use of MCS systems in adult patients.

## 3. Epidemiology of Myocarditis

The prevalence of myocarditis in the general population ranges from approximately 10 to 100 per 100,000 individuals. Furthermore, an estimated worldwide incidence of about 1.8 million cases per year has been reported [4]. The scarce accessibility of cardiac magnetic resonance imaging (CMR) and endomyocardial biopsy (EMB) across various countries can lead to difficulties in diagnosing myocarditis, causing inconsistent data and potential under-reporting of the disease in certain regions. A study conducted by Fu et al. examined changes in the incidence and prognosis of myocarditis in Sweden from 2000 to 2014. They were able to highlight an increase in cases from 6.3 to 8.6 per 100,000 individuals. This rise was supposedly attributed to enhanced diagnostic tools and improvements within the national healthcare system [5]. In light of these findings, a comparison of regional differences and different time periods appears even more difficult. Moreover, the emergence of the COVID-19 pandemic has significantly impacted the epidemiological landscape of myocarditis. Contemporary data suggest a high prevalence in patients diagnosed with SARS-CoV-2. In particular, male patients aged 30 years or older are affected, with an estimated yearly incidence of 57.2 to 114.0 cases per 100,000 following the infection [6]. In a meta-analysis, Voleti et al. found that unvaccinated patients had a sevenfold increased risk of experiencing myocarditis compared to those who had previously received SARS-CoV-2 vaccination [7].

### 3.1. Acute Myocarditis and Transition to Fulminant Forms

Within the spectrum of acute myocarditis (AM) lies a critical subset known as fulminant myocarditis (FM). It is characterised by the occurrence of potentially life-threatening acute HF. Patients suffering from FM often experience a quick progression towards cardiogenic shock (CS) and require immediate medical interventions. Several factors are known to highlight the transition from AM to fulminant forms and might act as ’red flags’ for the attending physician. Rapid onset of symptoms, including severe HF, CS, and the occurrence of malignant arrhythmias, can be viewed as precursors of aggravating situations subsequently requiring inotropes or MCS [3]. Furthermore, the occurrence of left bundle branch block (LBBB), premature ventricular contractions, decreased QRS amplitude, and ventricular tachycardia are reported to mark unfavourable progressions [8]. Regarding the transition towards FM, highlighting the mentioned factors holds particularly true in the absence of ischemic or pre-existing cardiomyopathies [9].

Epidemiological data on AM, in particular FM, appear scarce. Acute presentations seem equally distributed amongst male and female patients and appear more common in younger adults [10]. Based on the 2019 Global Burden of Disease Report, AM has an estimated rate of 6.1 cases per 100,000 in men and 4.4 per 100,000 in women aged between 20 and 44 years [11]. Furthermore, the prevalence of FM among patients suffering from AM was reported between 5% and 10%. Considering these estimations, the disease constitutes a rare yet highly challenging condition. Data from an international registry of 16 tertiary centres in Europe, the US, and Japan identified patients with AM and stratified them based on fulminant and nonfulminant presentations. Over the time span of 18 years, 220 patients were included. All patients presented with left ventricular systolic dysfunction, and 165 cases were classified as FM. Individuals diagnosed with fulminant forms exhibited significantly increased rates of mortality (ranging from approximately 12 to 62% under MCS, Table 1) and heart transplantation regarding both short- and long-term outcomes [12].

The recent COVID-19 pandemic had an impact on myocarditis in general but also affected the subset of FM. In a systematic literature review, Maya et al. identified 73 cases of FM in COVID-19-positive patients. Both males and females were almost equally distributed, and the median age was reported to be 45 years. The authors describe a comparably high necessity of inotropes and MCS paired with a high mortality rate of 27.7% [33]. Treatment options for myocarditis vary depending on the severity of disease and the underlying cause of myocarditis. Besides rest and symptomatic therapy, severe cases may require advanced cardiac support or heart transplantation [34]. In selected critically ill patients, prompt intervention with MCS systems is crucial in mitigating the development of haemodynamic instability [9]. Specifically, short-term interventions like ECMO or axial flow pumps can confer a vital time frame for proper diagnosis and the start of targeted treatment approaches. Moreover, permanent VAD systems can provide long-term solutions in patients without adequate myocardial recovery and serve as a bridge to recovery or transplantation [35,36]. The available anti-inflammatory treatment options for FM depend on the underlying aetiology of the condition. Diagnosis is primarily based on EMB, and histopathological findings are interpreted in accordance to the Dallas criteria [37]. However, issues such as sampling errors, variation in expert interpretation, and overlap with other inflammatory manifestations have led to discussions on the validity of these criteria [38]. Lymphocytic, giant cell, eosinophilic, and other forms of myocarditis were identified as histological subtypes of FM [39].

### 3.2. Indication and Timing of Short-Term MCS

Evidence-based recommendations regarding MCS in FM are scarce. The use of short-term options, including ECMO, intra-aortic balloon pumps (IABPs), and axial flow pumps, has been reported [15,28]. However, there is no clear consensus on the appropriate timing for initial MCS in the particular setting of myocarditis. Therapeutic decisions are often based on the degree of haemodynamic stability, metabolic and organ function, local procedures, and the general availability of mechanical support systems. Within an expert consensus document, Ammirati et al. summarised contemporary evidence and identified factors associated with a high probability of MCS requirement. The authors emphasised the clinical presence of cardiogenic shock, a left ventricular ejection fraction (LVEF) of less than 30%, and life-threatening arrhythmias as significant predictors of MCS necessity. In the presence of these factors, referral to a specialised centre, EMB, and temporary MCS are to be considered [9]. A position statement by the working group on myocardial and pericardial diseases (European Society of Cardiology, ESC) outlined similar recommendations. Patients displaying life-threatening manifestations of HF should be transferred to specialised units capable of extensive haemodynamic monitoring and EMB. Furthermore, in patients with haemodynamic instability, MCS systems may serve as a bridge to recovery or heart transplantation [2].

More recent evidence has been reviewed by the current ESC guidelines for the diagnosis and treatment of acute and chronic heart failure (published in 2021 and updated in 2023). Although not specific to FM, general recommendations are given for acute HF (AHF) and CS [40,41]. The guidelines recommend using MCS strategies selectively in specialised centres with multidisciplinary expertise regarding the implantation and management of circulatory support systems [42]. A ‘standardised team-based approach’ including early MCS implantation and close monitoring of invasive haemodynamics, laboratory markers indicating end-organ damage, and serial lactate measurements was reported to potentially improve outcomes [43,44]. The IABP-SHOCK-II trial revealed no tangible differences in 30-day mortality comparing optimal medical therapy and IABPs in CS patients following acute coronary syndromes with early revascularisation [45]. While not being investigated in all aetiologies of shock, the use of IABPs as a bridging option was assigned a class of recommendation IIb and a level of evidence C in patients with CS (class of recommendation III, level of evidence B in patients following myocardial infarction). Despite these recommendations, IABPs might improve ventricular unloading and coronary circulation. Other short-term MCS systems were evaluated more favourably in this context, with a class of recommendation IIa and a level of evidence C [40]. Moreover, the ESC guidelines particularly highlight the use of VA-ECMO in FM and other conditions causing pronounced CS [46]. Although only tested in infarct-related CS, the ECLS-SHOCK trial may question the efficacy of early ECMO implantation in unselected patient cohorts if there is no definitive therapy after bridging [47]. Considering the potentially reversible nature of FM, temporary MCS strategies should be prioritised in the early stages over permanent solutions.

**Figure 1:**
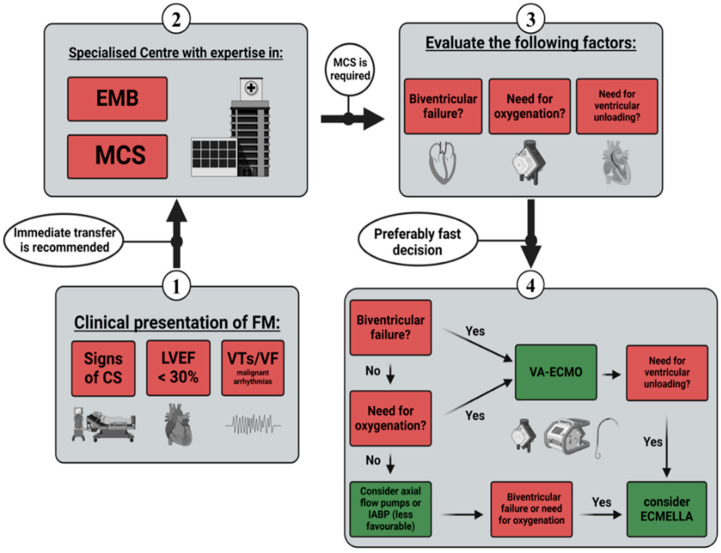
Management sequence for short-term MCS. CS—cardiogenic shock; LEVF—left ventricle ejection fraction; VT—ventricular tachycardia; VF—ventricular flutter; EMB—endomyocardial biopsy; MCS—mechanical circulatory support; IABPs—intra-aortic balloon pumps; VA-ECMO—venoarterial extracorporeal membrane oxygenation.

An analysis by Pahuja et al. uncovered trends in the epidemiology of myocarditis, CS, and the associated use of MCS in the United States from 2005 to 2014. They reported an increasing incidence in myocarditis paired with a drastically increased prevalence in CS (6.94% in 2005 vs. 11.99% in 2014) and required MCS (4.5% in 2005 vs. 8.6% in 2014). Contrary to these findings, in-hospital mortality remained unchanged (4.43% of total admissions over the study period), which might reflect the benefits of increased MCS utilisation. Moreover, within the observed time period, the usage of all MCS systems except IABPs increased significantly [48]. This particular finding can be interpreted in favour of short-term MCS, like ECMO or axial flow pumps, and may support the current ESC guideline recommendations regarding bridging strategies in CS [40]. According to available evidence in FM, the median time from the onset of AHF to ECMO implantation was reported between 13 and 15 h [14,18]. Previous studies on other aetiologies of CS suggested better outcomes with earlier implantation [49,50,51]. Axial flow pumps can serve as a treatment option for patients experiencing isolated left ventricular failure and for those without the need for supplemental extracorporeal oxygenation (or decarboxylation) support. Annamalai et al. conducted a study on the initial management of patients suffering from FM utilising Impella™ devices. Of the 34 patients included, 10 individuals required additional MCS, whereas complete recovery was observed in 15 cases without further support [28]. Randomised controlled trials (RCTs) comparing different MCS strategies in the early management of FM are lacking. Therefore, only assumptions and eminence-based opinions regarding the efficacy of various MCS systems are available. Based on the highlighted recommendations and supporting evidence, we created a diagram proposing a management sequence for short-term MCS in FM patients (Figure 1).

### 3.3. Outcomes in Fulminant Myocarditis with Short-Term MCS

Analysing the available evidence for short-term MCS in FM, consistent patterns appear to be present. Most patients were young to middle-aged adults with a median age of 31 to 51 years. VA-ECMO was most commonly used, with reported survival rates ranging from 38% to 87.5% at discharge (outlined in Table 1). According to the majority of the displayed studies, the survival rates for FM are generally higher than 60%. This indicates better outcomes in comparison to other causes of CS. In a study of 850 patients with suspected AM, Nunez et al. found that the hospital discharge rate of 65.1% was significantly higher than the 41% in an all-comer collective [29]. This finding underlines the importance of short-term MCS in haemodynamically unstable patients and emphasises the potentially reversible nature of the disease. Outcomes in FM associated with COVID-19 seem comparable to other aetiologies. Tonna et al. reported a 49% rate of survival to discharge, whereas Ammirati et al. found a survival rate of 78.5% after 120 days [31,32]. The data regarding IABPs and Impella™ show promising results in the context of survival and weaning success [10,28,32]. However, an underlying selection bias towards less critical patients without the need for additional oxygenation and biventricular failure cannot be ruled out. Moreover, in light of the latest recommendations for CS, IABPs can be viewed as less favourable compared to other short-term MCS systems [40]. Comparing central versus peripheral VA-ECMO, Tadokoro et al. highlighted better outcomes in patients with peripheral cannulation [24]. Whether this finding was driven by procedural differences or is attributed to the selection of patients without the need for ventricular unloading remains unanswered. 

### 3.4. Ventricular Unloading in Short-Term MCS

Although VA-ECMO implantation may be necessary for haemodynamic stabilisation in critically ill patients, it invariably increases left ventricular afterload. Without proper venting strategies, the resulting dilation of the left ventricle (LV) may cause pulmonary oedema as well as impaired myocardial regeneration due to haemodynamic and inflammatory pathomechanisms [52]. These adverse changes may be of particular importance in FM, as it constitutes a disease primarily driven by inflammation. Therefore, reducing wall stress through unloading may subsequently reduce the inflammatory response and improve ventricular recovery [53]. Implantation of axial flow pumps can potentially achieve this goal, and multiple case reports highlighted positive results in the short-time use of LV-Impella™ in FM [54,55,56]. Compared to different surgical venting strategies utilising VA-ECMO and cannulation of the left atrium (LA) or the LV, percutaneous Impella™ implantation may prove to pose a lower risk of bleeding or the occurrence of thromboembolism. Comparing Impella™ and ECMO treatment in patients with CS, Lamarche et al. reported comparable rates of 30-day mortality and hospital discharge. However, arterial thrombus formation and the requirement for blood products were found to be statistically less frequent in patients receiving Impella™ [57]. Overall, VA-ECMO and Impella™ treatment for FM were found to result in similar survival to discharge rates of approximately 60% [10,28].

Considering the advantages and disadvantages of both treatment methods, a combination appears feasible in selected cases. This approach, known as “ECMELLA”, combines potent haemodynamical support with the possibility of additional oxygenation and ventricular unloading [58]. Considering the recommendations for CS, a combination of VA-ECMO and Impella™ appears more favourable compared to VA-ECMO and IABPs [40]. In a large series of patients, Pappalardo et al. compared ECMELLA and VA-ECMO in patients suffering from CS (mixed aetiologies including CPR, STEMI, and PCI). Individuals treated with ECMELLA displayed a significantly reduced hospital mortality paired with an increased rate of successful bridge to recovery or subsequent therapy. Moreover, no tangible difference in major bleeding events was reported when comparing both groups [59]. In another study, it was discovered that left ventricular unloading reduced mortality among CS patients on VA-ECMO despite higher complication rates [60]. Although these findings are promising, confirmation in a collective exclusively comprised of FM patients is lacking. Recently, a new concept emerged providing biventricular support in FM using two Impella™ systems [61]. This approach, known as BI-PELLA, has been utilised successfully in several cases and might be of use in patients with biventricular failure without the need for additional oxygenation [62,63,64]. There are no clear recommendations on the timing of ventricular unloading. The EARLY-UNLOAD trial, which included 10% of patients with FM, found no difference in 30-day mortality between early and conventional unloading via left atrial cannulation in CS patients requiring VA-ECMO [65]. Decisions regarding the necessity of cardiac venting and the choice of MCS system are commonly based on clinical presentation and individual factors. The potential need for additional oxygenation, occurrence of biventricular failure, and ventricular volume overload are crucial points to consider in this context.

### 3.5. Weaning Strategies and Transition to Long-Term MCS

FM represents a condition with potential myocardial recovery, and short-term MCS systems should be used initially. However, questions regarding potential weaning strategies and the optimal time for transition to long-term MCS or heart transplantation arise. There are currently no guidelines or recommendations that are based on sufficient evidence. Despite the associated procedural risks and potential long-term complications, timely transplantation represents a feasible option for patients on MCS without significant weaning progress. In their study, Hsu et al. enrolled 75 adults suffering from FM who required VA-ECMO. Three patients received successful heart transplantation and survived to discharge [15]. In a study conducted by Ting et al., six patients with previous MCS underwent heart transplantation. Four of these patients survived to discharge [16]. Although heart transplantation appears to be a viable option in FM, not all patients in need of protracted haemodynamical support are eligible candidates for the procedure. Additionally, global and regional shortages of donor organs create the requirement for alternatives [66].

Before considering these options, weaning from short-term MCS systems should be attempted. Matsumoto et al. reported potential factors associated with ECMO weaning in 37 consecutive patients suffering from FM. Of these patients, 22 were successfully weaned, while 15 required further haemodynamic support. Subsequently, nine patients received VAD implantation. Ultimately only two individuals could be weaned off VADs and survived. The authors report significant differences in peak creatine kinase, CK-MB levels, the occurrence of arrhythmias, and left ventricular posterior wall thickness (LVPWT) as key predictors of weaning success. Furthermore, peak CK-MB levels > 185 IU/L and LVPWT values > 11 mm were found to be significantly associated with weaning failure [19]. Similarly, Chou et al. reported high initial CK-MB levels and malignant arrhythmias as predictors of poor myocardial recovery [23]. The “TIDE” algorithm, introduced in 2021 by Tschöpe et al. [67], is a novel approach to weaning Impella™ patients with therapy-refractory CS (designed for myocarditis, amongst other aetiologies). The four utilised steps include transthoracic echocardiography (during full ventricular unloading), time-gated Impella™ weaning in accordance with haemodynamic measurements (and echocardiographic assessment at minimal flow), dobutamine stress echocardiography, and right heart catheterisation at rest and during exercise. Using the protocol, the authors report high rates of successful weaning (74.2%) from Impella™ in selected patients (without ECMO, fever, haemodynamic instability, and a euvolemic status). The residual 25.8% displayed unsuccessful weaning and were considered candidates for LVAD implantation [67].

**Figure 2:**
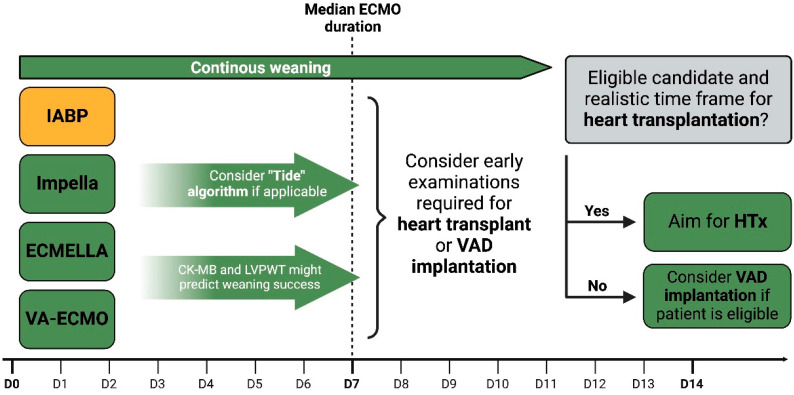
Transitioning from short-term to long-term MCS systems or heart transplantation. IABPs—intra-aortic balloon pumps; VA-ECMO—venoarterial extracorporeal membrane oxygenation; LVPWT—left ventricular posterior wall thickness; HTx—heart transplantation; VAD—ventricular assist device.

There are currently no clear recommendations regarding the duration of short-term MCS and the exact point at which patients should be transitioned to long-term MCS or heart transplantation. In their systematic review, Uil et al. reported a median ECMO support period of 6–7 days in patients suffering from refractory CS [68]. In a study of paediatric patients, Lee et al. found a high likelihood of transitioning to long-term treatment options if no recovery was observed within two weeks [69]. In the previously mentioned study by Hsu et al., patients displayed a median ECMO duration of 7 ± 5 days [15]. The previously reported evidence seems to favour early transplantation over the implantation of long-term MCS. However, not all patients are eligible candidates, and the shortage of donor organs paired with long waiting periods makes VAD systems a situational but viable choice. There are recent publications demonstrating the effectiveness of VAD implantation in patients suffering from FM [19,27]. In most of the reported cases, a left ventricular assist device (LVAD) was utilised and served as a bridge to transplant, recovery, or destination therapy. However, selected patients might require a bilateral ventricular assist device (Bi-VAD) [70]. Jaroszewski et al. even reported a patient with FM who underwent ECMO and was bridged to temporary Bi-VAD, followed by permanent VAD systems, and eventually, recovery [71]. Based on the highlighted publications, we propose a decision diagram for transitioning FM patients from short-term to long-term MCS systems or heart transplantation (Figure 2).

### 3.6. MCS and Additional Treatment in Lymphocytic Myocarditis

Lymphocytic myocarditis (LM) represents a subtype of myocarditis characterised by lymphocytic infiltration of the myocardium [72]. EMB should be performed to confirm the diagnosis and to distinguish between FM and inflammatory aetiologies characterised by infiltration of other cell lines. The clinical presentation ranges from mild symptoms to cardiogenic shock and life-threatening ventricular arrhythmias [73]. Viral infections are purported to be the most common cause of LM and can be detected in 30–40% of the affected patients [1]. Beyond the direct viral impact, cardiac injury may also emanate from an amplified immunological response termed molecular mimicry. This process involves the immune system erroneously targeting cardiac cells due to antigenic resemblance, thereby inducing myocardial damage [74]. Fulminant forms may require MCS systems as a bridge to recovery or long-term options, such as heart transplantation or VAD implantation. Particularly, rapid-onset variants may profit from immediate haemodynamic support, as spontaneous myocardial recovery was reported in some of these cases [75]. Furthermore, the MCS management of patients with fulminant LM should follow the discussed recommendations for FM and CS. Due to the inflammatory aetiology of the disease, multiple therapeutic approaches have been tested in the past. Particularly, the addition of corticosteroids and other anti-inflammatory agents were utilised with varying degrees of success. While therapy with prednisone alone did not lead to significant changes, combination with azathioprine resulted in improved myocardial recovery [76]. However, most of the reported studies used LVEF as an endpoint, but robust data on clinical outcomes, such as mortality, are lacking. Thus, there are no clear recommendations for the use of corticosteroids or immunosuppression in addition to general measures of haemodynamic stabilisation. Nonetheless, those substances are often used in clinical practice and may be viewed as an eminence-based therapeutic approach.

### 3.7. MCS and Additional Treatment in Giant Cell Myocarditis

Giant cell myocarditis (GCM) constitutes a rare but often fatal subset of FM [77]. This form predominantly affects young and middle-aged adults and presents with AHF as well as ventricular arrhythmias [78]. Histological findings show multifocal inflammatory infiltrates, including lymphocytes and multinucleated giant cells, which are typically located at the edge of the lesions. Due to this distinct histological presentation, EMB plays a crucial role in the diagnosis of GCM. However, several factors can impede the reliability of the procedure. Early samples may turn out negative, as giant cells typically appear after 7–14 days [79]. Furthermore, right ventricular EMB may be prone to the occurrence of sampling errors. Therefore, multiple biopsies, including samples of the LV, are often necessary for a definitive diagnosis of the disease [80]. Considering these implications, short-term MCS might confer a crucial time window of haemodynamic stability needed for proper diagnosis. Additionally, it is often used as a bridge to heart transplantation, which appears to be a viable, if not the most beneficial, long-term option for GCM [81]. Since the condition often affects both ventricles, biventricular MCS is required more often before transplantation in comparison to patients with cardiomyopathies caused by other aetiologies [82,83]. In addition to utilising haemodynamic support systems, early therapy with immunosuppressive agents is recommended [3]. Patients treated with cyclosporine, corticosteroids, and with or without an anti-CD3 antibody displayed a high survival rate after one year [84]. Due to the adverse side effects of anti-CD3 antibodies, later approaches used triple immunosuppressive therapy, including corticosteroids, cyclosporine, and azathioprine or mycophenolate mofetil [80,85]. A recent analysis highlighted the clear survival benefits of early immunosuppression compared to prior or exclusive treatment with MCS systems [86]. In conclusion, MCS systems are crucial for the haemodynamic stabilisation of patients suffering from fulminant GCM. However, early immunosuppressive therapy is essential to improve transplant-free survival or even achieve myocardial recovery [87].

### 3.8. MCS and Additional Treatment in Eosinophilic Myocarditis

Eosinophilic myocarditis (EM) is characterised by eosinophilic infiltration of the myocardium [88]. Amongst others, the condition has been linked to hypersensitivity reactions, hypereosinophilic syndromes, autoimmune disorders, infections, and active malignancies [89,90,91,92,93]. In a meta-analysis, Brambatti et al. characterised patients with histologically proven EM. They found that the median age was 41 years, 75.9% of them had peripheral blood eosinophilia, and there was a 22.0% correlation with asthma [94]. One of the most severe forms of EM is known as acute necrotising eosinophilic myocarditis (NEM). Although the condition is considered rare, it is associated with the rapid onset of CS, high rates of mortality, and the necessity of heart transplantation [95]. Similar to other aetiologies, such as GCM, EMB plays a crucial role in the diagnosis and further therapeutic management of EM and NEM. Notably, the most common cause of acute NEM appears to be drug hypersensitivity [3]. In both regards, short-term MCS may be of particular importance in providing haemodynamic stability until the diagnosis is made or potentially triggering medication can be stopped. There have been reports of ventricular thromboembolism occurring in individuals suffering from NEM [96,97]. This procoagulant potential must be considered while planning MCS strategies and managing anticoagulation. In contrast to GCM, high doses of corticosteroids appear effective in NEM [98,99]. Further approaches included the addition of mycophenolate mofetil and azathioprine [100,101]. There are multiple cases in which immunosuppression was administered, and MCS systems served as a bridge to recovery [100,102].

### 3.9. MCS and Additional Treatment in COVID-19-Associated FM

The emergence of the COVID-19 pandemic has brought forth a form of AM associated with SARS-CoV-2. Additionally, there is evidence supporting the occurrence of myocarditis following COVID-19 vaccination [103]. Unlike other forms of FM that are primarily defined by their histological features, COVID-19-associated AM displays heterogeneous histological presentations [32]. In both COVID-19- and vaccination-associated forms, LM appears to be the most commonly reported pattern, followed by eosinophilic and mixed infiltrates [33]. Ammirati et al. reported 10 fulminant cases that required temporary MCS systems with a median support time of 5 days. The most commonly used system was VA-ECMO, followed by IABPs [32]. Taking the simultaneous occurrence of COVID-19-associated respiratory failure into account, the use of MCS systems with the option for additional oxygenation seems feasible. However, cases without respiratory compromise could be treated with Impella™ alone [104]. The need for additional oxygenation as well as ventricular unloading may favour an approach such as ECMELLA, which has been used successfully in reported cases [105]. Guglin et al. reported higher rates of pulmonary infiltrates (chest X-ray) and shortness of breath in patients suffering from COVID-19 compared to vaccination-associated FM. Moreover, VA-ECMO was used more often in the COVID-19 group, possibly reflecting an increased oxygen demand. Despite these differences, overall mortality rates were comparably high in both groups [33]. As of yet, there are no clear treatment recommendations for COVID-19 and vaccination-related FM due to the novelty of the field. When EMB was available, most reported cases were treated based on their histological presentation. Furthermore, many patients received Remdesivir or Hydroxy-chloroquine [33]. Finally, Maunier et al. emphasised the possible therapeutic value of Anakinra in 7 paediatric cases of COVID-19-associated FM [106]. An overview of the specific subtypes of FM, the role of MCS, and additional treatment options are depicted in Table 2.

## 4. Discussion

Within the present review, we aim to shed some light on the role of MCS in the context of FM. Although the use of short-term MCS is recommended in haemodynamically compromised patients, there are no clear guidelines or recommendations on the timing or further escalation of the supportive therapy. We therefore sought to highlight recent publications and propose suggestions on these topics.

First, the details regarding the timing and criteria for implementing short-term mechanical circulatory support (MCS) were outlined. Guidelines and expert consensus statements on FM recommend the transfer to a specialised centre in case of life-threatening clinical presentation (signs of CS, LVEF < 30%, or malignant arrhythmias). The centres in question should have expertise in EMB and implantation as well as the management of MCS systems [2,9,40,41,42]. Factors such as local procedures and regional availability may play a role in selecting short-term circulatory support systems. The ESC guidelines recommend considering VA-ECMO in patients with FM and other conditions causing severe CS [40,41]. Although not specifically tested in all aetiologies of shock, The IABP-SHOCK-II trial led to a less favourable assessment of the IABPs compared to other short-term MCS systems [40,45]. However, both the ECMO-CS and ECLS-SHOCK trials may generally put the use of VA-ECMO in unselected patients into question [47,107]. Axial flow pumps such as Impella™ may pose an option for patients with CS in need of ventricular unloading. Comparably high rates of myocardial recovery under Impella™ support suggest effectiveness in the setting of FM [28]. Nonetheless, in the presence of biventricular failure or the need for additional oxygenation, the use of VA-ECMO appears clearly more feasible [3]. Data in FM may be limited, but early implantation of MCS has shown better outcomes compared to other shock aetiologies [49,50,51]. Based on the highlighted evidence, we aimed to visualise a potential management sequence for short-term MCS in FM patients (displayed in Figure 1).

Moreover, strategies for ventricular unloading are discussed in this review. Insufficient venting of the LV might be associated with pulmonary oedema and impaired myocardial recovery [52]. Besides surgical venting strategies utilising VA-ECMO and central cannulation, Impella™ appears to be a viable alternative. Although Impella™ and ECMO treatment demonstrate similar 30-day mortality and hospital discharge rates, Impella™ treatment is associated with a lower occurrence of thrombus formation and a lower need for blood products. [57]. A combined approach, known as “ECMELLA”, covers ventricular unloading and other factors, including biventricular failure and the additional need for oxygenation [58]. Compared to ECMO, ECMELLA was found to be associated with reduced hospital mortality and an increased success rate in bridging to recovery or subsequent therapy [59]. In patients without the need for additional oxygenation, a combination of two Impella™ systems, called BI-PELLA, might be used [61]. Currently, there are no randomised trials comparing the effectiveness of these MCS strategies in the context of FM. As a result, no official recommendations have been published yet. In most cases, individual decisions are based on factors such as the need for additional oxygenation, biventricular failure, and the presence of ventricular overload.

Furthermore, we aimed to highlight potential weaning strategies for patients suffering from FM requiring MCS. As already reported, timely heart transplantation represents a feasible option in the absence of significant weaning progress [15,16]. However, ineligibility for the procedure or a shortage of donor organs may require an alternative, i.e., long-term MCS systems. In many reports, VAD systems were used as a bridge to recovery or transplantation, and patients with persistent biventricular failure were successfully treated with Bi-VADs [19,27,70,71]. It is recommended to consider early examinations for transplant and VAD evaluation to avoid subsequent delay in case of unsuccessful weaning. Elevated levels of CK-MB and increased LVPWT were found to predict weaning failure and can, in this context, be considered indicators of accentuated myocardial injury [19,23]. The recently published “TIDE” algorithm represents a novel weaning approach in patients with therapy-refractory CS and Impella™. Utilising the protocol, high rates of successful weaning were reported in selected patients [67]. Currently, there is no clear recommendation on the ideal duration for short-term MCS or the precise point at which patients should transition to long-term MCS or heart transplantation. Most of the reviewed studies displayed a median ECMO support period of about 7 days. Support periods of more than 14 days were associated with worse outcomes and a high likelihood of unsuccessful weaning [15,68,69]. In this regard, we illustrate the transition of patients with fulminant myocarditis (FM) from short-term mechanical circulatory support (MCS) to long-term MCS systems or heart transplantation in alignment with the evidence presented (displayed in Figure 2).

Finally, we summarised MCS and additional treatment strategies according to the underlying aetiology of FM. In LM, rapid-onset variants may profit from immediate circulatory support, as these forms often exhibit spontaneous myocardial recovery [75]. Due to the lack of reliable outcome data, there is no clear recommendation for the additional use of corticosteroids or immunosuppression in LM. However, these substances are often used in eminence-based therapeutic approaches. In contrast, the therapeutic regime of GCM heavily relies on triple combinations, including corticosteroids, cyclosporine, and azathioprine or mycophenolate mofetil [80,85]. Additionally, there is evidence for survival benefits of early immunosuppression compared to prior or exclusive treatment with MCS systems [86]. Nevertheless, diagnosis of GCM via EMB and the initiation of therapy heavily relies on the crucial time window provided by MCS systems. Similarities are reported for NEM, which appears to be commonly caused by drug hypersensitivity [3]. The time required for diagnosis and the identification of potentially triggering medication is often acquired through short-term MCS. Importantly, NEM seems to be associated with increased rates of ventricular thromboembolism [96,97]. Therefore, an adequate anticoagulation regime should be considered. Compared to GCM, high doses of corticosteroids appear to be effective in treating NEM [98,99]. Moreover, therapeutic approaches, including mycophenolate mofetil or azathioprine, are reported [100,101]. Lastly, we shed some light on the evidence regarding COVID-19- and vaccination-associated FM. COVID-19-caused cases displayed higher rates of pulmonary infiltrates, shortness of breath, and VA-ECMO implantation. Thus, no differences in mortality appear tangible between these patients and those exhibiting vaccination-associated forms [33]. Due to the mixed histological presentations of these conditions, an EMB-based therapeutic approach seems feasible.

## 5. Conclusions and Future Directions

FM remains a condition defined by very high mortality and morbidity. Rapid onset of CS and subsequent multiorgan failure make correct diagnostic assessment and immediate medical attention imperative. MCS systems play a pivotal role in affected patients, as short-term solutions may confer the time required for adequate diagnosis or myocardial recovery. Additionally, they serve as a bridge to heart transplantation or long-term options such as VAD implantation. There are no clear recommendations regarding the initial timing and choice of MCS system. Due to the coverage of biventricular failure and the option of additional oxygenation and decarboxylation support, VA-ECMO appears to be the most commonly used choice. Although not tested explicitly in the setting of FM, IABPs received a less favourable class of recommendation for CS based on the results of the landmark IABP SHOCK II trial. However, the mentioned data on weaning success may highlight possible contributions to left ventricular unloading and the improvement in coronary circulation in selected patients. Axial flow pumps such as Impella™ were successfully used in patients with FM and proved effective as a venting strategy. With novel weaning protocols for therapy-refractory CS, these systems appear to be a viable choice in selected patients. Further therapeutic approaches, including the “ECMELLA” and “BI-Pella” concepts, were successfully used in the setting of FM. Thus, further studies are needed to compare their effectiveness and to determine which patients particularly benefit from their use. There are few structured weaning algorithms for MCS systems in myocarditis. Most studies reported a median VA-ECMO support period of approximately one week. Prolonged use appears to be associated with weaning failure and the necessity for long-term mechanical support or heart transplantation. Moreover, markers of myocardial injury and tissue oedema might predict weaning success. EMB and the resulting histopathological classification bear high relevance for further management of patients suffering from FM. Concomitant treatment with corticosteroids and immunosuppressive agents critically affects myocardial recovery in distinct subtypes of myocarditis. This particularly holds true for COVID-19- and vaccination-associated forms, which were reported to be caused by mixed aetiologies. Initial treatment approaches in FM were primarily drug-centred. With the emergence of mechanical support systems, this changed to a combined “life support-based comprehensive treatment” regimen utilising MCS systems and immunomodulation. The resulting reduction in mortality rates marks steps in the right direction. Following short-term MCS, subsequent heart transplantation or VAD implantation appears increasingly essential. New generations of improved circulatory support systems may provide better short- as well as long-term options for patients with FM. Additionally, novel antibodies and targeted anti-inflammatory therapy will possibly shape the therapeutic landscape of myocarditis in the coming years. To our knowledge, there are currently no large, randomised trials on the horizon comparing different MCS strategies in FM. However, collaboration on international registries might help to uncover new aspects of CS [108].

## Figures and Tables

**Table 1:** List of studies on MCS in AM. VA-ECMO—venoarterial extracorporeal membrane oxygenation; IABPs—intra-aortic balloon pumps; HTx—heart transplant; VAD—ventricular assist device; FM—fulminant myocarditis.

Authors	Time Period	Patients (n)	Type of MCS	Median Age	Outcomes
Aoyama, N., et al. [13]	1989–2000	52	VA-ECMO	~48 years	57.7% survival and return to normal life
Asaumi, Y., et al. [14]	1993–2001	14	VA-ECMO	~38 years	71.4% were weaned and survived to discharge
Hsu, K.H., et al. [15]	1994–2009	75	VA-ECMO	~30 years	61% survival to discharge
Ting, M., et al. [16]	1994–2014	93	VA-ECMO	~42 years	50.1% transplant-free survival
Ishida, K., et al. [17]	1995–2010	20	VA-ECMO	~45 years	60% survival to discharge
Diddle, J.W., et al. [18]	1995–2011	147	mainly VA-ECMO	~31 years	61% survival to discharge (9 HTx)
Matsumoto, M., et al. [19]	1995–2014	37	VA-ECMO	~43 years	59% successfully weaned from VA-ECMO
Chang, J.J., et al. [10]	1997–2011	294	99 IABP/195 VA-ECMO	~45/41 years	81%/61% survival to discharge
Mirabel, M., et al. [20]	2002–2009	35	VA-ECMO	~38 years	68.6% survival to discharge
Wu, M.Y., et al. [21]	2003–2010	16	VA-ECMO	N/A	87.5% survival to discharge
Beurtheret, S., et al. [22]	2005–2009	14	VA-ECMO	N/A	65% survival to discharge
Chou, H.W., et al. [23]	2006–2018	88	VA-ECMO	~42 years	46.6% successful weaning and discharge
Tadokoro, N., et al. [24]	2006–2020	70	VA-ECMO cent. 48/periph.22	~44/50 years	62%/95% weaning from VA-ECMO (total cohort survival 5 years: 76%)
Mody, K.P., et al. [25]	2007–2013	11	3 VA-ECMO/8 Bi-VAD	~48 years	73% survival to discharge (2 permanent VAD)
Lorusso, R., et al. [26]	2008–2013	57	VA-ECMO	~38 years	72% survival to discharge
Saito, S., et al. [27]	2009–2015	25	23 VA-ECMO/2 t-VAD	~39 years	83.3% survival to discharge (6 permanent VAD)
Annamalai, S.K., et al. [28]	2009–2016	34	Impella (2.5, CP, 5.0, or RP)	~42 years	61.8% survival to discharge (15 weaned, 5 transferred, 1 HTx)
Nunez, J.I., et al. [29]	2011–2020	850	VA-ECMO	~41 years	65.1% survival to discharge
Danial, P., et al. [30]	2015–2018	47	VA-ECMO	~46 years	37.9% survival to discharge
Tonna, J.E., et al. [31]	2020–2021	88	VA-ECMO	~48 years	49% survival to discharge (FM + COVID-19)
Ammirati, E., et al. [32]	2020–2021	10	IABP/VA-ECMO	~38 years	78.5% survival after 120 days (FM + COVID-19)

**Table 2:** Overview of myocarditis subtypes. VA-ECMO—venoarterial extracorporeal membrane oxygenation; SARS-CoV-2—severe acute respiratory syndrome-related coronavirus 2; MCS—mechanical circulatory support.

Subtype	Histopathology	Aetiology	MCS	Additional Treatment
Lymphocytic myocarditis (LM)	Mononuclear cellular infiltrates (T lymphocytes)	Virus/autoimmune-mediated (molecular mimicry)	Particularly rapid-onset variants may profit from immediate haemodynamic support, as spontaneous myocardial recovery was reported in some cases	No clear data on corticosteroids and immunosuppressive therapy, but often used as an eminence-based therapeutic approach
Giant cell myocarditis (GCM)	Multifocal inflammatory infiltrates, including lymphocytes and multinucleated giant cells	Unknown, potentially associated with autoimmune disorders	Survival benefits of early immunosuppression compared to prior or exclusive treatment with MCS systems. However, MCS is often required to provide haemodynamic stability needed for diagnosis	Corticosteroids and immunosuppressive therapy recommended
Eosinophilic myocarditis (EM)	Eosinophilic infiltration of the myocardium	Hypersensitivity reactions, hypereosinophilic syndromes, autoimmune disorders, infections, and active malignancies	Importance in providing haemodynamic stability until diagnosis is found or triggering medication can be stopped	Corticosteroids (±immunosuppressive therapy) recommended
COVID-19-associated myocarditis	Heterogeneous histological presentations	Infection with SARS-CoV-2	According to histological presentation. Often requirement of VA-ECMO due to additional need for oxygenation	According to histological presentation. Sometimes Remdesivir or Hydroxy chloroquine, no clear data
